# Effectiveness of Integrated Google Classroom, Reciprocal Peer Teaching and Flipped Classroom on Learning Outcomes of Research Methodology: A Natural Experiment

**DOI:** 10.7759/cureus.16176

**Published:** 2021-07-04

**Authors:** Meenakshi Khapre, Smita Sinha, Pawna Kaushal

**Affiliations:** 1 Community and Family Medicine, All India Institute of Medical Sciences, Rishikesh, Rishikesh, IND

**Keywords:** covid-19, online learning, google classroom, flipped classroom, reciprocal peer teaching (rpt)

## Abstract

Background

To maintain physical distancing for reducing the spread of COVID-19, online learning appears to be a viable option to carry on teaching and learning.

Aim

The aim of study was to assess the effectiveness of integrated flipped classroom and reciprocal peer teaching (RPT) using Google Classroom as a learning management system (LMS) for teaching and learning, a module of Research Methodology. We also aimed to assess learner’s satisfaction.

Methods

An educational interventional study was conducted with 17 students enrolled in the Master of Public Health course, All India Institute of Medical Sciences, Rishikesh, for one month. As per protocol development and integration were conducted and validated pre- and post-tests were held for assessment of knowledge and skill component. Class normalized learning gain was used as objective measure for improvement in knowledge and skill. Students' feedback was collected using a structured questionnaire at the end of module.

Results

Mean test scores of knowledge and skill, improved significantly from 26.4 (11.95) to 33.64 (6.63) and 17.88 (5.7) to 62.76 (18.18) respectively. Class average normalized gain for knowledge and skill was 30.28% and 55.67, respectively. Students agreed that online learning imparted good understanding, at comfortable pace, opportunity for interaction. Students felt poor network affected their learning.

Conclusion

The study concluded that flipped classroom and RPT integrated with Google Classroom is an effective intervention.

## Introduction

The emergence of the coronavirus disease in 2019 (COVID-19) and the subsequent pandemic have disrupted education worldwide. Classroom teaching has been suspended to maintain physical distancing and reduce the risk of COVID-19 transmission. In the current situation, online learning appears to be a viable option for teaching and learning. At present, the education system is relying heavily on technology, mainly driven by electronic learning (e-learning) [1]. E-learning, or online learning, provides us with the technology to deliver, support, and enhance teaching/learning and establish communication between learners and teachers [2].

Online learning platforms allow learners to control the content, pace, and environment of learning. Apart from functioning as a repository of e-learning resources, a learning management system (LMS) can track students’ performance and be used for small group teaching [3]. Google Classroom is an open-source LMS. Teachers can effectively create and collect assignments online, as Google Classroom weaves together Google Docs, Drive, and Gmail and automatically creates Drive folders for each assignment and student [4].

In a study conducted by Dash in 2019, students reported that Google Classroom provides better access to learning material and supplementary learning resources, immediate feedback, and learning outside of the class environment than traditional lectures [3]. It can also give the students "easier and more effective access to a wider variety and greater quantity of information" with the help of a flipped-classroom approach [5,6]. Further, in the current study, the reciprocal peer teaching (RPT) approach is used, a form of collaborative learning where students of the same academic year switch their role as tutor/tutee. A systematic review confirmed that RPT improves the engagement, enthusiasm, and interest of learners if it is well planned and supported by faculty [7].

Research Methodology (RM) is a core module for the Master of Public Health (MPH) degree. Considering the problem of limited access to a high-speed network for many MPH students living in remote areas, Google Classroom was chosen as an LMS for teaching and learning the RM module. The educational goals of medical education technology are to facilitate basic knowledge acquisition, improve decision-making and team coordination, improve skills, and enhance learning satisfaction [8]. Keeping these goals in mind, the objectives for the present study were to assess the effectiveness of the integration of Google Classroom, the flipped classroom model, and RPT in terms of the average learning gain in knowledge and skill components related to RM. We also assessed the learner’s satisfaction with this integrated teaching-learning (T-L) method. 

## Materials and methods

**Figure 1:**
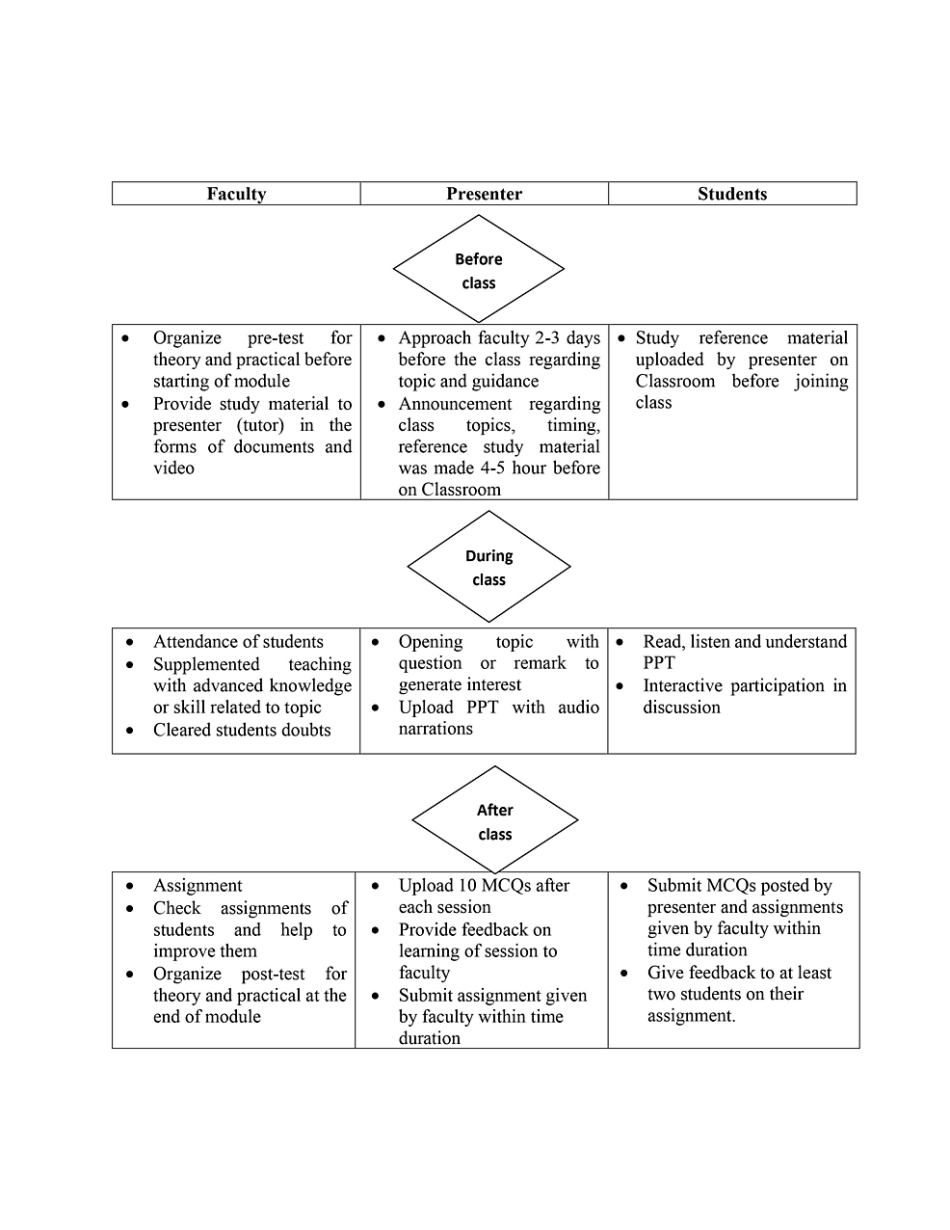
SOP for Google Classroom SOP: standard operating procedure; MCQ: multiple choice question

This educational interventional study was carried out at All India Institute of Medical Sciences, Rishikesh, among MPH students. Realizing the urgent need to shift the T-L process to virtual mode from physical classroom mode, we explored feasible available technologies for carrying out class in virtual mode. We intentionally selected Google Classroom. Before starting the online course, an orientation class was conducted to familiarize the students and other faculty members with this platform. To make learning synchronous, a two-hour T-L session was conducted five days a week with a judicious mix of narrated slideshows, learning material, quizzes, and a discussion board. Standards of procedure (SOP) were laid down for online classes using the flipped classroom and RPT approach via Google Classroom (Figure 1).

The RM module was mainly assignment-based and progressed gradually from basic introduction of RM and formulation of research questions to full protocol development. A daily assignment was posted on the assignment page of Google Classroom by a student, and this assignment was visible to all in the form of a shared Google Docs file. Each student had to give constructive feedback on at least two students’ work to qualify for daily attendance. Discussion boards in Google Classroom were used to maintain interaction among students. Pre- and post-module tests for the knowledge component were administered using Google Forms. The tests included 50 multiple-choice questions (MCQs; 20% recall and 80% analytical type). For the skill component, students were asked to create a protocol before and after the module completion. We (authors MK and SS) were blinded to the timing of the protocols and graded them anonymously. Grading was done using the attached proforma in the appendix, and the maximum achievable mark was 100.

After the module, students’ feedback via Google Forms was gathered using a structured questionnaire containing eight positively stemmed items scored on a Likert scale, two dichotomous yes/no questions, and two open-ended questions. On the 5-point Likert scale, 5 meant students strongly agreed and 1 meant they strongly disagreed with the statement given in the feedback form.

Statistical analysis

Data were entered in Microsoft Excel 2012 and double-checked for errors. The Wilcoxon signed-rank test was used for differences in the pre- and post-test scores of the knowledge and skill components.

Individual actual gains, Gi (Gi = post-test score − pre-test score), were calculated for all students, and absolute gain was calculated using the following formula: ∆ = average Gi/maximum score achievable.

The relative gain, expressed as a percentage, was calculated as follows:

C = average Gi/pre-test score

A class-average normalized gain (g) of 30% was taken to define the minimum value at which educational intervention could be regarded as effective [9,10], with Normalized gi defined as the average actual gain divided by the maximum possible gain; gi was calculated as:

Normalized gi= [post-test % − pre-test %]/[100 − pre-test %]

The class-average normalized gain (g) was calculated by adding individual single-student normalized gains (gi) and dividing by the total number of students. However, there was a possibility that the post-test score could be lower than the pre-test score (i.e., negative gain); in that case, the negative gain was replaced by zero. Students with this result were not accounted for in the denominator for the calculation of average gain.

Ethical considerations

Ethical approval was obtained from the Institutional Ethical Committee of the All India Institute of Medical Sciences, Rishikesh, India.

## Results

Seventeen students in their first and second semesters of the MPH degree participated in online classes to complete the RM module through Google Classroom. All 17 students took both the pre-test and the post-test. Fourteen of these students were women, and three were men.

The mean test scores for knowledge improved significantly, increasing from 26.4 (11.95) to 33.64 (6.63; p = 0.003). Absolute gain and relative gain for knowledge were 14.47% and 27.39%, respectively. The individual average normalized gain was 23.58%. Two students had negative gains for the knowledge component, so the average normalized gain was calculated as 30.28% (n = 15). Mean test scores for skill improved significantly, increasing from 17.88 (5.7) to 62.76 (18.18; p = 0.000). The absolute and relative gains were 44.88% and 250.95%, respectively. None of the students had negative gain; therefore, the class-average normalized gain was 55.67% (n = 17) (Table 1).

**Table 1:** Comparison of pre- and post-test scores of knowledge and skill learning gain among students

	Pre-test scores mean (SD)	Post-test scores mean (SD)	Wilcoxon signed-rank test Z(p)	Absolute gain	Relative gain	Class normalized gain
Knowledge	26.4(11.95)	33.64(6.63)	-3.012 (0.003)	14.47%	27.39%	30.28%
Skill	17.88(5.7)	62.76(18.18)	-3.626 (0.000)	44.88%	250.98%	55.67%

**Figure 2:**
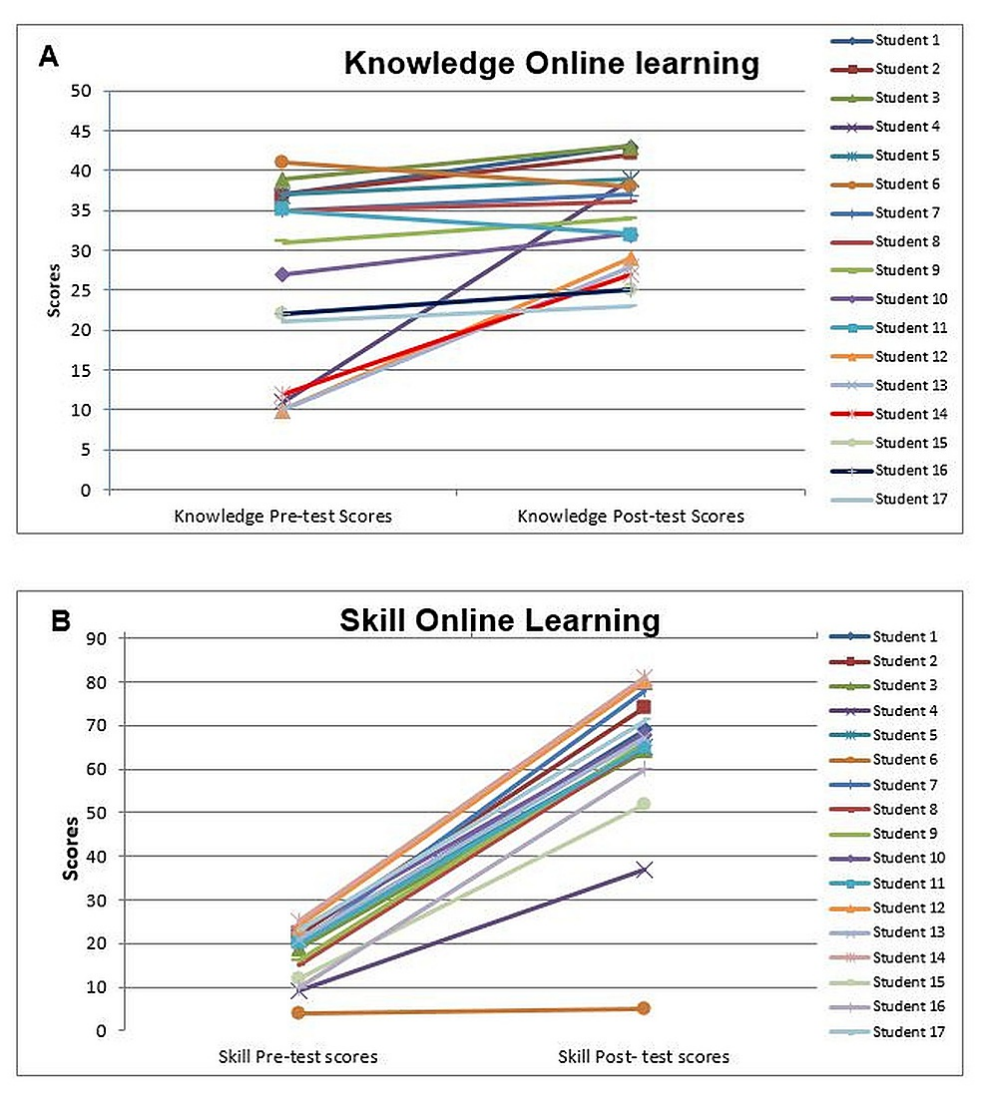
Pre- and post-test knowledge and skill score of students

Assessment of online learning (knowledge) was done by comparing pre- and post-test scores. These plots showed improvement (positive slopes), no change (horizontal lines), or deterioration (negative slopes) at the end of the course. Out of 17 students, 15 students (88.23%) showed improvement in the post-test scores compared with the pre-test scores. For online learning (skill), pre- and post-test score plots showed improvement (positive slopes), no change (horizontal lines), or deterioration (negative slopes). All the students showed improvement in practical post-test scores when compared to pre-test scores (Figure 2).

**Figure 3:**
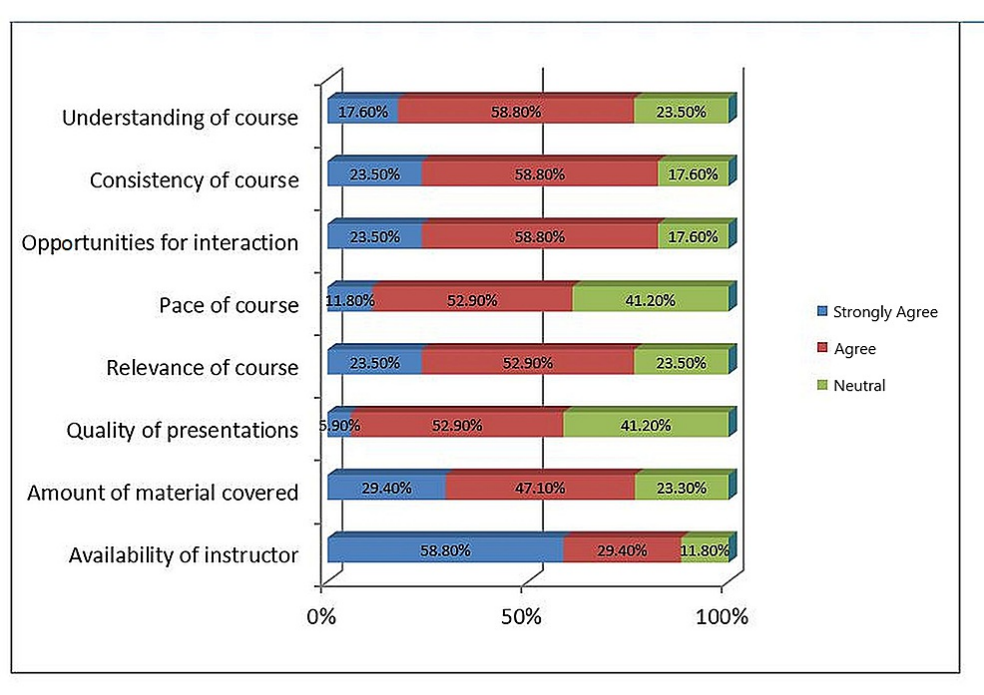
Feedback from students for online learning

Figure 3 presents distributions of the feedback module through the Google Classroom platform. Most students agreed with the comfortable pace of learning (11), the ready availability of instructor (15), consistency with learning objectives (LOs) (14), good understanding (13), relevance to LOs (13), and adequacy of material covered (13). Only 10 students agreed that the quality of presentations was excellent, and the rest were neutral on this point. None of the students disagreed with any of the selected criteria for satisfaction.

More than half the students (10) stated that they attended the module at a comfortable and well-timed pace. Almost all the students said that the course had nicely narrated presentations and interactive discussions in the presence of a moderator. Nine students said quality material was provided in the form of documents and videos. All students said that quizzes were an exciting part of the course. Nine students reported network and electricity issues that affected performance in exams. More than one-third of students (7) were not satisfied with RM skills (Table 2).

**Table 2:** Feedback comments on the strength and weakness of the online RM module * reported by more than 30% of students RM: Research Methodology

Questions	Comments (N=17)
Q.1 What were the strengths of this e-learning course?	Learned practical applications; active course kept students engaged; attended in comfort; learnt the course at well-timed pace*; course was consistent, scalable and offers personalization; full availability of moderator and good healthy discussions*; good interaction with teacher and students*; enough time for better understanding*; nicely narrated presentations*; the students themselves do most of the work, so it gives a sense of responsibility; extra course material provided during discussion in the form of documents and videos*; quizzes were interesting; assignments were relevant to the topics
Q.2 What were the weaknesses of this e-learning course?	Some presentations were not satisfactory; network and electricity issues sometimes*; faced difficultly during online exam*; lack of flexibility; no face to face interaction; sometimes doubts were not wholly solved during discussions; sometimes moderator was unavailable; It was more of textual knowledge and lacks a bit in improving the practical skills*; sometimes difficult for students who were not good in technical knowledge; need to type on discussion board, no audio option

## Discussion

The present study is the first of its kind in public health education because it has integrated different teaching styles, including the flipped model and RPT using Google Classroom as an LMS, to give an enhanced experience to students undergoing RM module training. We tried to make learning synchronous using discussion boards so that interactivity is maintained and issues solved in real time.

The present study showed the effectiveness of incorporating flipped classroom teaching and RPT in Google Classroom to improve knowledge and skill outcomes in RM for public health specialists. The average learning gains were 30% and 55% in knowledge and skill, respectively. Three students (3/17) had a negative gain in the knowledge component, which was due to technical issues. The feedback from students also pointed to good understanding, consistency, and relevancy of the module, as well as space for learning delivered via the virtual classroom. This finding is similar to that of a study by Dash, who reported that Google Classroom is a better tool for sharing learning resource material and examining students’ progress [3]. Students appreciated the opportunity for interaction through the discussion board and felt a sense of responsibility toward learning, as also reported by Dash [3]. A recent systematic review in medicine, nursing, and pharmaceutical education showed that flipped classrooms are as effective as lectures [11-13]. Ramadhani et al. found that the average mathematics learning outcomes of students taught using flipped problem-based learning with the help of Google Classroom were better than those taught using conventional learning methods. Moreover, a significant increase in learning was observed compared with conventional instruction [14].

Learning by teaching is an age-old concept. Incorporating the element of teaching in learning improves learning outcomes. Peer teaching has been used in higher education, including medical education. In this study, we found that the RPT approach maintained student enthusiasm in learning and improved the presentation quality. The present study findings were consistent with a systematic review on RPT reporting that it enhances understanding and retention of the topic, improves course grades, inculcates self-directed learning, and improves knowledge and skills [7]. Kassab et al. compared student-led seminars (SLSs) with faculty-led seminars and found that the tutorial atmosphere, decision making, and supportive feedback from group leaders were better in SLSs. In the present study, students reported that the discussion was open and the environment was friendly [15]. However, some authors have suggested that although RPT is effective, it provides an unreliable quality of teaching from peers; thus, they emphasized the need for special training before the RPT approach [16,17]. In this study, RPT was closely supported by a faculty member, as evident in feedback from students (58% opined that moderators were always available) and good-quality narrated presentations by students. Interestingly, higher attendance (100%) was reported in virtual classrooms, which was similar to the finding reported by Kogan et al. [17] 

Technology-related challenges were the most common challenges faced in the virtual mode of learning. Despite trying to maintain interaction through the discussion board, 13 students out of 17 felt isolated. The module was mainly assignment-based, but students felt it was more theoretical. Students felt that a few complex topics could be more effectively learned through face-to-face interaction. In addition, students perceived that typing on the discussion board hindered learning, and the integrated audio option could have saved time; this option could be more useful if used in future programs.

The study has limitations in terms of sample size because we could include only 17 students. It was a natural experiment, and the students enrolled in this study were the students who took the RM course during the study period. Although we tried to make the module enjoyable, and it was assignment-based, some students failed to participate actively in the course.

## Conclusions

We conclude that integrating a flipped classroom and RPT using Google Classroom effectively improved the knowledge and skill of students learning RM. It is feasible to integrate different teaching styles through the virtual mode. Students were satisfied with their learning outcomes, and acceptance was high because the approach provided them with a flexible environment. We recommend virtual instruction should be supplemented with weekly face-to-face interaction in the future. Effective learning through a virtual classroom, depends on student readiness and motivation to learn, which should be assessed before imparting this integrated approach and tailored to learners’ style of learning. Network connectivity is a major issue; government should take the initiative to provide reliable network at a low cost to students. 

## Appendices

Proforma provided to the students

Name of student:

Protocol title:

Rubric grading for protocol (Table 3):

**Table 3:** Rubric for protocol PICOT: patient/problem, intervention, comparison, outcome, time; SMART: specific, measurable, achievable, relevant, time-bound

Sr no		Not at all written (0)	Does not meet expectations (1)	Approaching expectations (2)	Meet expectations (3)	Exceeds expectations (4)
1	Title appropriate; not lengthy, study design and place mentioned					
2	Introduction includes key concepts and relevant background information.					
3	Literature review (comprehensive and clear)					
4	Rationale and relevance (finer criteria)					
5	Hypothesis					
6	Research question (specific, researchable questions that have the potential to add to the current body of knowledge (PICOT)					
7	Objective (SMART)					
8	Sufficient detail to determine that methodology is sound (quality, the validity of data is maintained)					
9	Sufficient detail to carry out the experiment or replication, including subject, setting, procedure, etc.					
10	Appropriate study design is chosen with details mentioned					
11	Specific independent and dependent variables are appropriate to the objective					
12	Quantitative components are appropriately added					
13	Detailed framework of data analysis					
14	Dummy tables address the objective					
15	Ethical consideration mentioned					
16	Gnatt chart					
17	Citation done by referencing manager					
18	References (one or other style) by manager					
19	Questionnaire / schedule (variety of questions added )					
20	Logical flow of question					
21	Questions framed in a correct manner					
22	Absence of ambiguous questions					
23	Appropriate in length and relevant to the objective					
24	Overall writing skill and clarity					
25	Grammar, punctuation, and spelling					
	Total marks					

Feedback
